# Sex Differences in Primary HIV Infection: Revisiting the Role of TLR7-Driven Type 1 IFN Production by Plasmacytoid Dendritic Cells in Women

**DOI:** 10.3389/fimmu.2021.729233

**Published:** 2021-08-27

**Authors:** Jean-Charles Guéry

**Affiliations:** Institut Toulousain des Maladies Infectieuses et Inflammatoires (INFINITY), Université de Toulouse, INSERM, CNRS, UPS, Toulouse, France

**Keywords:** HIV, innate immunity, sex bias, type I IFN, plasmacytoid dendritic cells, Toll-like receptor 7

## Abstract

Plasmacytoid dendritic cells (pDCs) produce type I interferon (IFN-I) during HIV-1 infection in response to TLR7 stimulation. However, IFN-I-signaling has been shown to play opposite effects in HIV-1 and SIV infection. TLR7-driven type I interferon production in pDCs is higher in women than in men due to the cell-intrinsic actions of estrogen and X-chromosome complement. Indeed, TLR7 is encoded on the X-chromosome, and the *TLR7* gene escapes the X-chromosome inactivation in immune cells of women which express significantly higher levels of TLR7 protein than male cells. Following HIV infection, women have a lower viremia during acute infection and exhibit stronger antiviral responses than men, which has been attributed to the increased capacity of female pDCs to produce IFN-α upon TLR7-stimulation. However, a deleterious functional impact of an excessive TLR7 response on acute viremia in women has been recently revealed by the analysis of the frequent rs179008 c.32A>T SNP of *TLR7*. This SNP was identified as a sex-specific protein abundance quantitative trait locus (pQTL) causing a difference in the TLR7 protein dosage and effector function in females only. T allele expression was associated with a lower TLR7 protein synthesis, blunted production of IFN-α by pDCs upon TLR7 stimulation, and an unexpectedly lower viral load during primary HIV-1 infection in women. In the present review, the author will revisit the role of TLR7-driven pDC innate function in the context of HIV-1 infection to discuss at what stage of primary HIV-1 infection the TLR7 rs179008 T allele is likely to be protective in women.

## Introduction

Sex differences in the acquisition and pathogenesis of HIV-1 disease between men and women have long been reported in epidemiological studies, reviewed in (1, 2). Women have half the plasma viral load men have in both acute/primary and chronic HIV-1 infection in the absence of treatment (3–5), while such difference has no influence on time to AIDS progression between sex (6). A more robust type I interferon (IFN-I) response during primary HIV infection in women relative to men has been hypothesized to explain the initial plasma viremia differences between sex (2). Assertion supported by the observation that following viral replication adjustment, women had a higher expression of IFN stimulated genes (ISG) in T cells and higher levels of T cell activation as compared to men (7, 8). The strength of the innate and adaptive immune responses of women is generally greater than those of men (9), and in part explained by the qualitative and quantitative differences in plasmacytoid dendritic cells (pDCs) innate function between sex (10). Indeed, pDCs are specialized in the production of IFN-I through TLR7 signaling and play an important role in bridging innate and adaptive immunity in the context of HIV-1 infection (11). Cell-associated and, less efficiently, cell-free HIV-1 virions signal through TLR7 to induce IFN-I and other pro-inflammatory cytokines and chemokines (12, 13). An elevated plasma IFN-I level characterizes the acute and late chronic phases of the infection (14, 15). Studies of macaques infected with simian immunodeficiency virus (SIV) point to pDCs as the critical IFN-I producers in this process (16, 17). Likewise, in humanized mice, depletion of pDCs prior to HIV-1 infection boosted HIV-1 replication and abolished serum IFN-I elevation and the expression of ISG (18). However, pDC-derived IFN-I production was also associated with the capacity of HIV-1 to induce cell death, immunosuppressive pathways, and immunopathogenesis during the acute phase of primary infection (16–18). Likewise, studies in the SIV infection model have also provided a contrasting view suggesting that the beneficial or detrimental role of pDCs and IFN-I may vary depending on the timing (19) or the site of infection (20). Indeed, in the course of intrarectal SIV infection, an initial IFN-α2a administration prevented systemic infection (19), supporting a beneficial effect of IFN-I during acute infection (21). Moreover, an intravaginal infection in female macaques pointed to a deleterious role of the epithelium-innate immune cells axis, characterized by an early pDC recruitment and activation locally underneath the epithelium layer of the endocervix (20). In the chronic phase of the infection, a continued IFN-α2a treatment in the course of intrarectal SIV infection accelerated CD4^+^ T cell depletion, disease progression, and death from AIDS (19), suggesting a detrimental effect of IFN-I as the disease progresses (21, 22).

The frequency of pDCs producing IFN-α is significantly higher in women than in men upon TLR7 engagement (8, 23, 24), which correlates, during primary infection, with the clinical differences in the course of HIV-1 infection together with a greater expression of ISG (7, 8). It has been proposed that these sex differences in the responses to HIV-1 hinge in a large part on the sex bias in IFN-I production by pDCs (25). However, the role of TLR7-driven pDC activation and IFN-I production in HIV-1 patients is not clearly defined. Recently, we examined this question in terms of the frequent single nucleotide polymorphism (SNP) of *TLR7* rs179008 c.32A>T, which was found to selectively impair the TLR7 protein expression and TLR7-driven production of IFN-α by pDCs in women, but not in men (26). The minor T allele of rs179008 is especially frequent among European populations, where 30%–50% of women are homozygous or heterozygous carriers, and common worldwide except in East Asia. Contrary to the expectation, we observed that the blunted interferogenesis in women who were carriers of the T-allele was associated with a lower viremia and reduced frequency of symptoms at acute HIV-1 infection when exploring the French PRIMO cohort which is composed of women infected by sexual transmission (26). Thus, we demonstrated for the first time that the *TLR7* rs179008 is a sex-specific protein expression QTL (pQTL) of potential significance in the control of HIV-1 viral infection specifically in women.

In the present review, the author will revisit the role of TLR7-driven pDC innate function in the context of HIV-1 infection to discuss at what stage of primary HIV-1 infection. Based on the recent study (26), the author will also propose that the higher propensity of female pDCs to produce IFN-I in response to TLR7 stimulation is unlikely to contribute to the sex differences in an acute HIV-1 infection.

### Biological Sex is an Important Driver of Toll-Like Receptor 7 Mediated Type 1 Interferon Production by Plasmacytoid Dendritic Cells

The plasma HIV-1 RNA load at the “set point” (4–6, 27) as well as the associated HIV-1 DNA reservoir cells (28, 29) are lower in women compared to men. The most classically suggested hypothesis to explain this gender bias is the difference in the innate immunity observed between men and women (25). Indeed, the frequency of IFN-α-producing pDCs is higher in women compared to men after the stimulation with TLR7 ligands (8, 10, 24). One of the mechanisms explaining this enhanced activation threshold of pDCs in female is related to estrogens (24, 30). Interestingly, beside the female sex hormone effect, it was also shown that X-chromosome dosage contributed to this sex biased response of pDCs (31). TLR7 is encoded on the X-chromosome, and we recently showed that the TLR7 gene escapes X-chromosome inactivation in female immune cells (32). This mechanism is responsible for a significant increase in the amount of TLR7 protein in the leukocytes of women compared to men (32), and probably contribute to the enhanced capacity of pDCs to express higher levels of TLR7 mRNA and to transcribe higher levels of IFN-α/ß mRNA at a steady state and in response to TLR7 ligands (33). Thus, both estrogen-signaling and X-linked genetic factors contribute to the stronger functional response of female pDCs (24, 30, 31).

### Sex-Specific Impact of the Toll-Like Receptor 7 rs179008 Protein Expression Quantitative Trait Locus on Plasmacytoid Dendritic Cells Innate Functions

**Figure 1:**
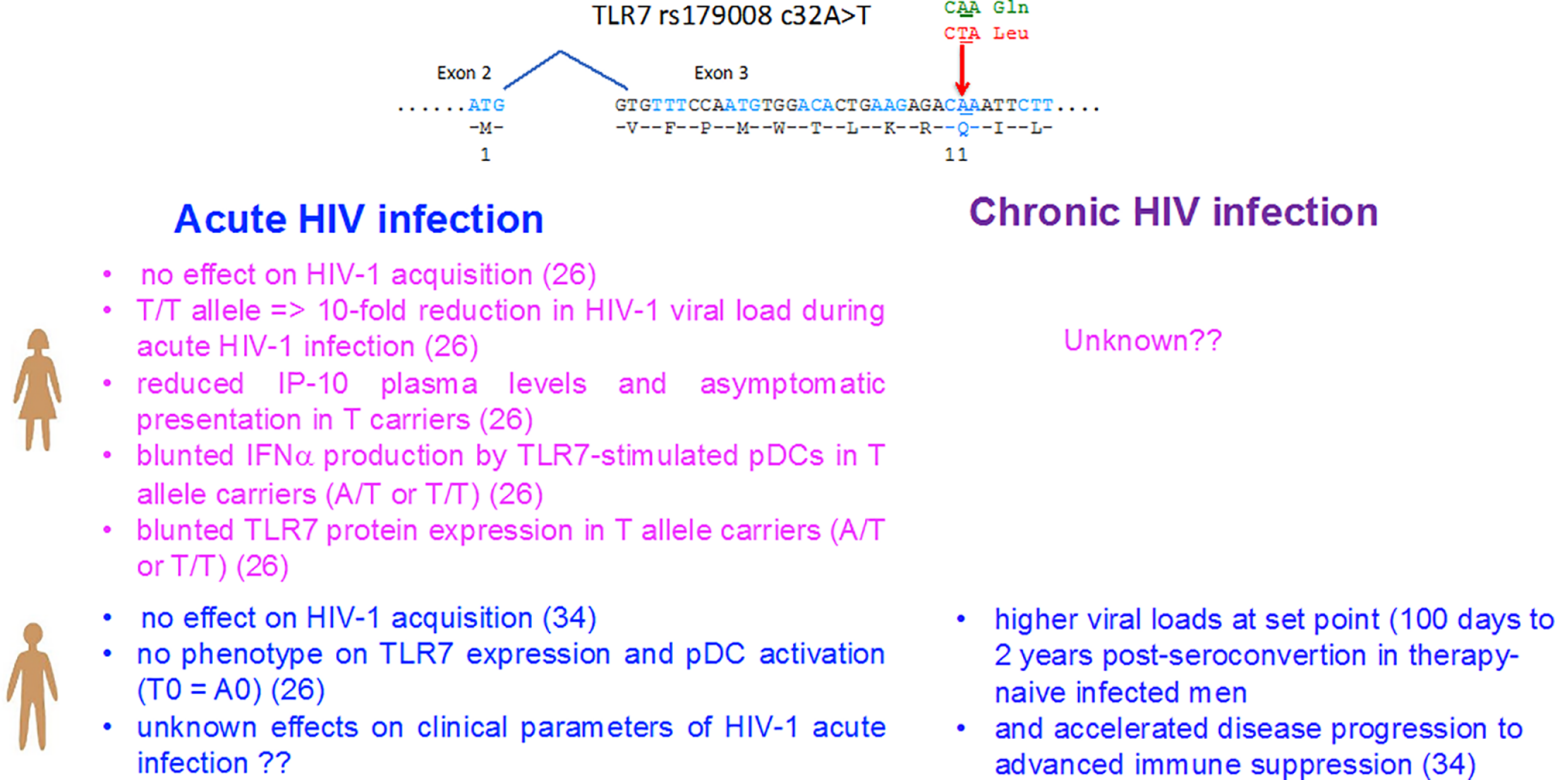
Impact of the sex-specific pQTL rs179008 c.32A/T SNP on TLR7-driven responses and parameters of HIV-1 infection in women and men. The rs179008 SNP is located in the leader sequence of *TLR7* at position 32 in codon 11, and introduces an amino acid substitution from Gln to Leu. As shown in ref (26), the minor allele rs179008 T of *TLR7* impairs type I interferon production by pDCs in response to TLR7 ligands, but only in women. The sex bias in pDC function mirrors a genotype-dependent drop in TLR7 protein expression in female leukocytes, consistent with the notion that TLR7 dosage in pDCs determines type I interferon secretion. Primary HIV-1 infected women carrying the rs179008 T allele exhibited an almost 10-fold reduction in the RNA viral load, as well as reduction in cell-associated DNA, plasma IP-10, and symptomatic presentation at diagnosis (26). In ref (34), it was initially reported that men carrying the T alleles exhibited higher viral loads at set points compared to A0 men and showed accelerated reduction in CD4^+^ T cell counts (see **Box 1**). The allelic frequency of rs179008 in the European 1000 Genome project population is 0.76 (A) and 0.23 (T). The genotype frequencies found in our cohorts in average were: Female AA (62%), AT (33%), TT (5%), Male A0 (73%), T0 (27%).

The female predominance in the TLR7-driven production of IFN-I by pDCs represents a robust phenotype that distinguish the innate immunity between men and women, then it is not surprising that genetic mutations have been selected during the evolution to counterbalance this effect. Among them, the NM_016562.3:c.32A>T (dbSNP rs179008) polymorphism of TLR7 is a missense SNP substituting a leucine (c.32A) for a glutamine (c.32T, p.Gln11Leu) in the leader sequence of the TLR7 protein. This SNP was previously identified by Oh and coll (34) as a risk factor for being infected by HIV-1 in c.32T allele carriers in women but not in men, whereas an accelerated deterioration of CD4^+^ T-cell counts was also reported in men carrying the T0 (c.32T) genotype (**Figure 1**) (34). Not tested in women by Oh and coll (34), the functional impact of rs179008 c.32T on protein expression and in TLR7-driven pDC responses was recently examined in both sexes (26). Interestingly, the stronger TLR7-driven IFN-α responses of pDCs from women as compared to men (8, 23, 24) was further magnified when all carriers of the c.32T allele of TLR7 were excluded from the analysis (26). Mechanistically, c.32 T allele was identified as a sex-specific pQTL found to determine the lower TLR7 protein expression in female leukocytes, which mirrored its negative functional impact on the TLR7-driven production of IFN-α by pDCs from women but not from men (**Figure 1**). Unusually, this nonsynonymous SNP affects the signal peptide which controls protein dosage at the translational level through the mRNA sequence itself, rather than through the alteration of the signal peptide function. Indeed, evidence was provided for a translation rate-limiting role of Leu_11_ codon in allele T leader peptide. Leu11 corresponds to the first rare codon in the path of the ribosome consistent with the 5′ to 3′ direction of ribosome scanning, and we hypothesized that the ribosome occupying codon 11 may sterically hinder the recognition of the translation initiation codon by the next ribosome upstream, given the 20- to 30-nucleotide footprint of a ribosome (35) and the 32- to 39-nucleotide interval between consecutive ribosomes in tightly packed polysomes (36). Local translation efficiency depends on the abundance of aminoacyl-tRNAs with compatible anticodons, and this is positively correlated with the relative usage of synonymous codons (37). It is conceivable that the effect of the codon substitution on translation efficiency might be felt only in female cells. Differences in codon usage bias have been described between male-, female-, and non-sex-biased genes in insects (38), avians (39), and fish (40), possibly arising from a selective advantage linked to the translation efficiency in either sex. Whether the sex dependency observed in the TLR7 quantitative protein phenotype for rs179008 could be mediated by sex-differences in the regulatory mechanism of translation will deserve further investigations.

### Impact of Toll-Like Receptor 7 rs179008 Protein Expression Quantitative Trait Locus in Primary Human Immunodeficiency Virus 1 Infection in Women

Because the T allele of rs179008 pQTL affects TLR7 protein expression and in turn TLR7-driven production of type I IFNs by pDCs in women, but not in men, its direct contribution on the clinical parameters of HIV-1 infection at the initial/acute phase of the disease was investigated in women (**Figure 1**). Contrary to the study of Oh and coll (34), a meta-analytical combination of two separate case-control settings, restricted to Caucasian women, did not support a significant genetic association of rs179008 with HIV-1 acquisition through the sexual route in women (26). In a striking contrast, in HIV-1 primary infected women from the French ANRS PRIMO cohort, the carriage of the rs179008 T allele was associated in the initial/acute phase of the disease with a decrease in viremia, a reduction in HIV-1 DNA associated cells, and lower plasma concentrations of the interferon alpha/gamma-induced protein 10, IP-10 also known as CXCL10 (**Figure 1**). Symptomatic presentations of acute HIV-1 infection were also less common in T/T homozygous women, where the RNA viral load is decreased by an average of eight to nine times (26). Patients with detectable plasma IFN-α exhibit elevated levels of IP-10, and plasma IP-10 can be used as a surrogate of the IFN-I signature to demonstrate a positive correlation between IFN-I with viral load (41–43), and with a rapid disease onset during primary HIV-1 infection (43, 44). Thus, the higher frequency of asymptomatic clinical presentations at diagnosis for the T/T homozygous women implies a trend for a delayed onset of the symptoms of acute infection in this subgroup (26). Others frequent TLR7 SNPs were also investigated, such as the frequent intronic tag SNP, rs179012, previously associated with HIV-1 set-point viral load, especially in females (45), and a frequent functional SNP in the 3′ UTR of *TLR7*, rs3853839 in genetic association with the risk of developing systemic lupus erythematosus in males (46). None of these SNPs alone were found to be associated with the clinical parameters of a primary HIV-1 infection. Together, the genetic association studies reinforced the conclusion that rs179008 was a functional polymorphism of *TLR7* in its own right (26), and strongly suggested that effector functions associated with TLR7 activation might exert dominant deleterious effects on viral load during acute HIV-1 infection in women (**Figure 1**).

### Evidence for a Negative Impact of the Toll-Like Receptor 7-Driven Innate Immunity and Type I Interferons Production During Natural Human Immunodeficiency Virus/Simian Immunodeficiency Virus Infection

**Figure 2:**
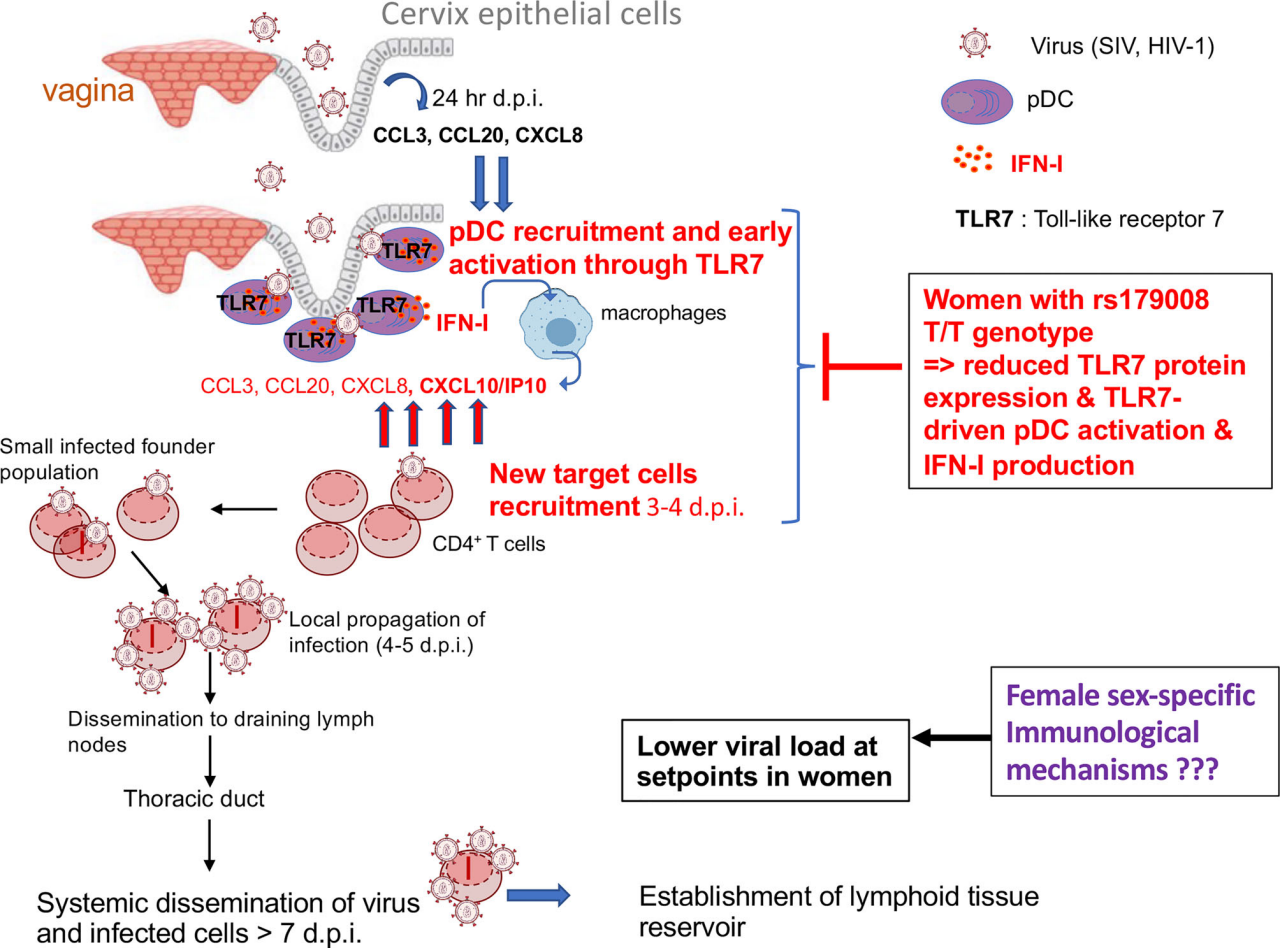
Model of infection and dissemination of HIV-1. Cervical epithelium in the female reproductive tract responds through unknown mechanisms to exposure to the SIV inoculum by producing CCL3, CCL20, and CXCL8. pDCs are the first innate cells to be recruited by day 1-3 post-infection by epithelial-derived chemokines [and other innate cells such as macrophages at later time points (48)], which can be activated by viral particles which can penetrate the mucosal barriers. IFN-I released by pDCs can induce IP-10/CXCL10 production by macrophages and TLR7-activated pDCs themselves produce CCL3, CCL5, and IP-10/CXCL10 to create a focal chemokine gradient beneath the epithelium which recruits CCR5 expressing CD4^+^ T cells to establish productive infection, creating a feed forward loop to sustain pDCs recruitment and further increases in CD4 T-cell targets (47, 48).

HIV-1/SIV transmission primarily occurs in the female reproductive tract (FRT) in a small founder population of cells at mucosal sites followed by a period of intense replication (47) (**Figure 2**). The local expansion is fueled by the influx of new target cells recruitment, including CD4^+^ T cells, through a gradient of chemokines produced by innate immune cells such as pDCs (20) and monocytes (48). These sequential events in early mucosal responses lead to CD4^+^ T cell recruitment (**Figure 2**). This starts with the release of chemokines, such as CCL20, by the epithelial mucosa sensitized by SIV, followed by a massive recruitment of CCR6^+^ pDCs which becomes shortly activated to produce IFN-I, IP-10/CXCL10 and other CCR5^+^ T cell-attracting chemokines (20) (**Figure 2**). The virus expands locally using the influx of target cells and then disseminate *via* lymphatic drainage to genital draining lymph nodes where more viruses are quickly produced in the T cell areas (47). SIV then spread through the thoracic duct to the lymphatic tissues resulting in an intense viral replication, resulting in high levels of plasma viremia and systemic dissemination of virus to lymphoid organs (47, 49).

Sex differences in the acquisition rates of HIV-1 infection have been observed with a two-fold higher risk for women compared to men following a serodiscordant encounter (50). This differences in transmission are likely a consequence of a unique anomatological characteristic of the FRT as compared to penile or rectal surfaces (51). Indeed, this anatomic difference is a critical domain of sex differences relevant to HIV infection with the paradox that an activated mucosal immunity at the cervicovaginal mucosa lowers the barrier to HIV infection (51). This was confirmed in female macaques exposed with TLR7 or TLR9 agonist prior SIV infection, with the aim to trigger anti-viral immunity, showing that an endosomal TLR engagement is effective to enhance infection and viremia (52). In favor of an immune quiescence model of protection, it was further reported that HIV-negative exposed sex workers exhibited a depressed mucosal immunity as compared to uninfected controls (53). Indeed, genital tract inflammation during early HIV-1 infection predicts an increased risk for HIV acquisition (54), and higher viral load at set point and CD4^+^ T cell depletion in women. Thus, as suggested by others (19, 20, 47), infection through the FRT may depend on IFN-I signaling for CD4^+^ T cell recruitment and virus propagation. By contrast, infection through the rectal mucosa, which contains many resident CD4^+^ T cells can be protected by the antiviral properties of IFN-I (19). Thus, depending on the sites of entry rectum *versus* FRT antiviral mediators such as IFN-I might be protective or deleterious, respectively (19).

### How Can Sex-Differences in the Toll-Like Receptor 7-Driven Innate Plasmacytoid Dendritic Cell Function Contribute to Sex-Bias in Human Immunodeficiency Virus Infection?

Based on all these considerations, the author propose that an enhanced TLR7 protein expression in a subset of women (c.32A allele carriers), associated with an enhanced innate sensing of viral components through TLR7, characterized by a higher IFN-I production by female pDCs could contribute to promote HIV-1 mucosal transmission and/or replication in the female genital tract (**Figure 2**). This may explain the strong protective effect we observed in all clinical parameters of HIV-1 acute infection in women carrying the rs179008 T/T alleles, which reduces TLR7 protein expression in all immune cells and negatively impact the production of IFN-α by pDCs in response to HIV-1 ssRNA (26) (**Figure 2**). Thus, the higher functional responsiveness of woman pDCs appears deleterious in the general context of primary HIV-1 infection, suggesting that the better control of viral load and the HIV-1 reservoir in primary infected women compared to men is not due to the enhanced capacity of female pDCs to produce IFN-I during the early-stage of HIV-1 infection. This necessarily implies that the better control of viremia at setpoints in women is probably not a direct consequence of the anti-viral properties of IFN-I during the acute phase of infection as previously suggested (25) but could be due to a direct effect of estrogen-signaling on HIV-1 replication (55) or other unknown mechanisms (**Figure 2**).

By contrast, the detrimental role of IFN-I in chronic infection is supported by many studies and probably explains the faster progression to AIDS in women than in men. In chronic HIV infection, women have a higher level of CD8^+^ T cells activation after adjustment for viral loads, that correlates with an enhanced functional response of pDCs, suggesting that the sex-bias in the TLR7-driven production of IFN-I by pDCs could contribute to the inflammatory mechanism responsible for the more rapid disease progression seen in women (8). A deleterious role of pDC-derived IFN-I production has been illustrated by the lymphocytic choriomeningitis virus (LCMV) murine model of persistent viral infection. Here, blockade of IFN-I signaling even during the acute phase of infection facilitated a long-term viral clearance (56, 57). In humanized mouse and SIV models, pDC-depletion or IFN-I receptor (IFNAR) blockade, respectively, rescued human T cell depletion and function despite elevated HIV-1 or SIV replication (18, 58). In these models, the upregulation of ISG, high level T cell activation, and T cell dysfunction persisted during antiretroviral therapy (ART), and blocking IFN-I signaling not only enhanced T cell recovery (59, 60) but also reduced the HIV-1 reservoir (59, 60), and delays HIV-1 rebound after ART discontinuation (59). Whether sex influences the therapeutic response to IFNAR blockade in chronic HIV will deserve further investigation.

## Concluding Remarks

Although the results obtained with the rs179008 SNP might appear counter intuitive regarding previous works by others supporting a beneficial role of IFN-I-signaling and pDCs by controlling viral replication during acute infection in SIV and humanized mouse models (18, 19, 61), they illustrate the “hard to predict” nature of manipulating IFN-I or innate immunity during the course of HIV-1/SIV infection (19, 20, 22, 47, 62). In women carrying the rs179008 T allele, although not totally inhibited, such early mechanisms initiated by pDCs-derived IFN-I would be less operating resulting in a significant inhibition of viral spreading and expansion at the early stage of infection (**Figure 2**). The consequences of this SNP at the later stage of infection and on chronic HIV-1 disease outcome appears protective in men (**Box 1**), through unknown mechanisms, and will, however, deserve further investigations in women (**Figure 1**). In light of the beneficial effect of IFN blockade on the HIV reservoir in humanized mouse models (59, 60), it has been proposed that targeting IFN-I signaling will reverse HIV/ART-associated inflammatory diseases, rescue anti-HIV T cells, and reduce HIV reservoirs (22). IFNAR1-specific antibody is clinically safe and have been used in SLE patients which are preferentially women. Thus, taking into account the rs179008 SNP expression will be of particular interest, as one can speculate that women rs179008 T carriers would respond better to IFN-I blockade therapy than women expressing the frequent A allele only, in which TLR7 dosage and TLR7-driven pDC interferogenesis are at the highest. Together, it is therefore likely that the frequent rs179008 c.32.T pQTL of *TLR7* might be beneficial in women at all stages of HIV infection.

Box 1 ***TLR7* rs179008 and HIV-1 Infection in Men.**

In the study by Oh et al. (**Figure 1**), the TLR7 Gln11Leu polymorphism, however, was associated with a more severe HIV disease progression in HIV-1 infected men, while not tested in women (34). This study failed to confirm the functional impact of this SNP neither on the IFN-I production by pDCs from men, nor the TLR7 protein expression in male immune cells at a steady state (26). The impact of this SNP on primary acute infection in men will deserve further investigation. Although, in healthy donor cells, TLR7 protein expression was mostly found in pDCs, monocytes, and B cells, and it was also detected, at lower levels, in CD4^+^ T cells (63). In the latter subset, TLR7 engagement has been shown to induce human CD4^+^ T cell anergy and to promote T cell susceptibility to HIV-1 infection (63). Thus, the mechanism by which rs179008 SNP of TLR7 regulates HIV-1 disease progression in men is currently unknown.

## Author Contributions 

The author confirms being the sole contributor of this work and has approved it for publication.

## Funding

This work has been supported by grants from the French National Agency for Research on AIDS and Viral Hepatitis (ANRS, EP-53 study), SIDACTION, and Conseil Régional Occitanie-Midi-Pyrénées.

## Conflict of Interest

The author declares that the research was conducted in the absence of any commercial or financial relationships that could be construed as a potential conflict of interest.

## Publisher’s Note

All claims expressed in this article are solely those of the authors and do not necessarily represent those of their affiliated organizations, or those of the publisher, the editors and the reviewers. Any product that may be evaluated in this article, or claim that may be made by its manufacturer, is not guaranteed or endorsed by the publisher.

## Abbreviations

HIV-1, human immunodeficiency virus 1; SIV, simian immunodeficiency virus; pDCs, plasmacytoid dendritic cells; TLR7,Toll-like receptor 7; IFN-I, type 1 interferon; ISG, interferon stimulated genes; ART, antiretroviral therapy; pQTL, protein expression quantitative trait locus; IFNAR, type I IFN receptor.
